# *IL-1 β* gene (+*3954 C/T,* exon 5, rs*1143634*) and *NOS2 (exon 22)* polymorphisms associate with early aseptic loosening of arthroplasties

**DOI:** 10.1038/s41598-022-22693-0

**Published:** 2022-11-01

**Authors:** Esteban López-Anglada, Julio Collazos, A. Hugo Montes, Laura Pérez-Is, Imanol Pérez-Hevia, Sergio Jiménez-Tostado, Tomás Suárez-Zarracina, Victoria Alvarez, Eulalia Valle-Garay, Víctor Asensi

**Affiliations:** 1 Traumatology Department, Hospital Universitario Central de Asturias, Oviedo, Spain; 2 Infectious Diseases Section, Hospital de Galdacano, Vizcaya, Spain; 3 Biochemistry and Molecular Biology Department, University of Oviedo School of Medicine, Oviedo, Spain; 4 Infectious Diseases Unit, Infectious Diseases Section, Hospital Universitario Central de Asturias, University of Oviedo School of Medicine, Avda Roma s/n, 33011 Oviedo, Spain; 5 Molecular Genetics Section, Hospital Universitario Central de Asturias, Oviedo, Spain; 6 Group of Translational Research in Infectious Diseases, Instituto de Investigación Sanitaria del Principado de Asturias (ISPA)., Oviedo, Spain

**Keywords:** Genetics, Immunology, Biomarkers, Diseases, Medical research, Pathogenesis, Rheumatology, Risk factors

## Abstract

Aseptic prosthetic loosening (APL) and prosthetic joint infections (PJI) are frequent complications of hip and knee implants. Polymorphisms of cytokines and nitric oxide (NO), key inflammatory molecules in APL and PJI pathogenesis, could explain individual susceptibility to these complications. Three cytokines (*IL-1-a, IL-1-β, TNF-α*) and two nitric oxide synthase (*NOS2, NOS3*) genes polymorphisms were genotyped in 77 APL and 117 PJI patients and 145 controls with aseptic hip or knee implants that were implanted for > 16 years. Plasma cytokines and nitrate-nitrite (NOx) levels also were measured. The *TT* genotype and *T* allele of (+*3954 C/T, exon 5, rs1143634*)* IL-1β* polymorphism were more frequent in APL patients compared to controls (P = 0.03 and P = 0.02, respectively). No genotypic associations in PJI patients were observed. Plasma IL-6, TNF-α and NOx were significantly different between APL and controls (P < 0.0001). Plasma IL-1β and IL-6 were significantly higher in APL *T* allele carriers vs. non-carriers (P < 0.03). Knee implant (HR 2.488, 95% CI 1.307–4.739, P = 0.005), male gender (HR 2.252, 95% CI 1.121–4.525, P = 0.023), carriages of the *TT* genotype of the (+*3954 C/T) IL-1β* polymorphism (HR 3.704, 95% CI 1.274–10.753, P = 0.016) and *AA* genotype of the *(exon 22) NOS2* polymorphism (HR 3.509, 95% CI 1.266–9.709, P = 0.016) were independently associated with a shorter implant survival by Cox regression. No genotypic associations in PJI patients were observed. Genotyping of *IL-1β *(+*3954 C/T, exon 5, rs1143634*) and *NOS2 (exon 22)* polymorphisms could be useful as predictors of early hip or knee APL.

## Introduction

Aseptic prosthetic loosening (APL) is a major clinical problem that results in pain and thus the need for revision surgery^1–5^, but the pathophysiology behind this complication is unclear. However, proinflammatory cytokines play an important role in bone remodelling^6–8^. Interleukin-1 isoforms (IL-1α and β) and tumor necrosis factor-α (TNF- α) are potent stimulators of bone resorption by inducing osteoclastogenesis and studies have shown that cytokines are involved in hip and knee APL. Ex-vivo studies of cultured synovium from patients with failed prostheses produced high levels of IL-1, TNF-α and IL-6^9^. In situ hybridization of the same membranes showed high levels of IL-1β mRNA in macrophages^4^.

Genes encoding *IL-1α, IL-1β, TNF-α* and other cytokines are polymorphic, and the various alleles may have different translational efficiency, affecting cytokines production^10–13^. Associations between cytokines polymorphisms: (*-238 A/G*, *rs361525*) of *TNF-α*, (*-174 G/C*, *rs1800795*), (*-597 G/A*, *rs1800797*) and (*-572 G/C*, *rs 1800796*) of *IL-6*, (-*330 T/G*, *rs2069762*) of *IL-2*, and (*29 T/C*) of the transforming growth factor-β 1 (*TGF-β1*) have been reported to be risk factors for osteolysis after hip arthroplasty^14–17^. Polymorphisms of *IL-1α* and *β,* although closely associated with bone inflammatory and infectious diseases^11,18–20^, have not been studied in depth in APL. Late loss of bone after denture implant is associated with genetic polymorphisms of *IL-1-α (-889 C/T) rs1800587), IL-1-β (*+*3954 C/T, exon 5, rs1143634) and VNTR IL1RN, rs2234663*^21^.

Nitric oxide (NO), a free radical produced through the metabolism of arginine by the nitric oxide synthase (NOS), is crucial in bone metabolism. The NOS endothelial isoform (eNOS or NOS3) is constitutively expressed in bone, whereas inducible NOS (iNOS or NOS2) is expressed by bone cells in response to IL-1-β and TNF-α. The NO produced stimulates bone loss^22,23^. *NOS3* gene expression is regulated by inflammatory stimuli through the Akt-kinase pathway^24^. Knock-out mice for *NOS3* have marked defects in osteoblast function^25,26^.

Some *NOS3* polymorphisms have been linked to differences in NO blood levels or in protein expression in response to several stimuli, including the 27-bp repeat in intron 4, the *(-786 T/C,*) in the promoter region *(rs2070744*), and the missense (*E298D*) in exon 7 *(rs1799983*). These *NOS3* polymorphisms are associated with bone diseases^27,28^. Several polymorphisms of *NOS2*, such as the highly polymorphic CCTTT micro-satellite at the promoter region, the *G/A* substitution at position 37498, and G/A in exon 22 (iNOS 22), are associated with rheumatoid arthritis^29,30^. However, the association between *NOS3* or *NOS2* polymorphisms and APL has not been studied so far.

Despite careful management of preventive measures, prosthetic joint infections (PJI) can develop in up to 1.7% of primary hip and 2.5% of knee arthroplasties^31^. A functional variant of of *IL-1β (-511 C/T*, rs*16944*) is associated with increased susceptibility to PJI as are polymorphisms of the mannose-binding lectin (MBL) (-*550 C/G, rs11003125*, codon *54 A/G*, *rs1800450*), toll-like receptor 9 (*TLR9*) *(-1486 T/C, rs 187084*), and the vitamin D receptor (VDR) (*T/C, rs1544410*)^32,33^.

The aim of this study was to analyze a potential association between hip and knee PJI and APL and some polymorphisms of cytokines and NOS that were previously associated with inflammatory bone diseases, (*[-889 C/T] IL-1α, [*+ *3954 C/T, exon 5] IL-1β*, *[-308 G/A] TNF-α]), ([-786 T/C] NOS3* and [*exon 22, NOS2]*). We compared a group of APL and PJI patients with hip or knee implants to control patients who had neither complication after ≥ 16 years. A secondary aim was to study the expression of cytokines and *NOS* in the different genotypes by measuring IL-1β, IL-6, TNF-α and nitrate-nitrite (NOx) plasma levels in APL and PJI patients and controls.

## Material and methods

### Patients

We recruited patients who were admitted with APL of hip or knee for a second arthroplasty or with PJI for antimicrobial treatment at the Hospital Universitario Central de Asturias (HUCA) between January 2003 and April 2021. Surgical confirmation of the APL and a negative bacteriological culture of surgical samples were required for inclusion as APL. PJI patients were included if they fulfilled the 2013 Infectious Diseases Society of America PJI diagnostic criteria^34^. Cultures of surgical and sinus tract samples from PJI patients were collected.

The main indication for the primary arthroplasty was degenerative osteoarthritis in all the APL and PJI patients, and controls. Other diseases which could affect the bone metabolism, such as hyperparathyroidism, chronic kidney insufficiency, rheumatoid arthritis and other inflammatory arthropathies, steroids use, autoimmune diseases, and avascular necrosis of the femoral head, were excluded from the study.

Controls were individuals admitted to the HUCA to undergo a primary hip or knee arthroplasty since January 2003 and were followed ≥ 16 years until April 2022 or implant–unrelated death without developing APL or PJI.

Patients and controls were members of a homogeneous population; all were Europeans and residents of the same region (Asturias, Northern Spain). Each participant gave written informed consent for the study, which was approved by the Ethics Committee of the HUCA with the number 2020.113.

### Genotypic analysis

Genomic DNA was extracted from peripheral leukocytes following a salting-out method^35^. Polymorphisms of cytokines *([-889 C/T] IL-1α, rs1800587, [*+*3954 C/T] exon 5 IL-1β, rs1143634, and [–308 G/A]), TNF-α, rs1800629], NOS3 [-786 T/C], rs2070744, and NOS2 [exon 22*]) were determined by PCR (Table 1)^18,27^. The PCR results were confirmed by sequencing representative samples for each genotype of each polymorphism. PCR products were electrophoresed on a 2% low-melting agarose gel and the fragments were excised from the gel, purified with spin columns (DNA gel extraction Kit, Millipore, Billerica, MA, USA) and sequenced on an ABI Prism 310 Genetic Analyser (Applied Biosystems, Foster City, CA, USA).

**Table 1:** Oligonucleotide primer sequences, PCR conditions and restriction enzymes used for genotyping and sequencing of the different polymorphisms studied.

Gene	Polymorphism	Primers	PCR length (bp)	Annealing temperature (ºC)	Restriction enzyme
*TNF-α*	*-308 G/A*	Forward:5′-GCAATAGGTTTTGAGGGCCAT-3′ Reverse:5′-GGGACACACAAGCATCAAG-3′	147	58	*Nco I*
*IL-1α*	*-889 C/T*	Forward:5′-ATCACACCTAGTTCATTTCCTCTATTTA-3′ Reverse:5′-GATTTTTACATATGAGCCTTCCATG-3′	195	58	*Nco I*
*IL-1β*	+*3954 C/T, exon 5*	Forward:5′-CTCAGGTGTCCTCCAAGAAATCAAA-3′ Reverse:5′-GCTTTTTTGCTGTGAGTCCCG-3′	194	60	Taq^α^ I
*NOS3*	*-786 T/C*	Forward:5′-TGGAGAGTGCTGGTGACCCCA-3′ Reverse:5′-GCCTCCACCCCCACCCTGTC-3′	180	62	*Msp* I
*NOS2*	*exon 22*	Forward:5′-CTCCCGGGATCACACGCCCA**T**-3′ Reverse:5′-GCTGAATCTGAGTTGATGAACAGATG-3′	140	60	*Nco* I

The underlined bases in the primers differ from the original sequences and served to introduce a restriction site or to disrupt a natural restriction site within the primer sequence.

### Cytokines levels

Plasma cytokine levels (IL-1β, IL-6, TNF-α) were measured by ELISA kits from R&D Systems (R&D Systems Inc., 614 McKinley Place, MN, USA). Cytokine concentrations were calculated by comparing sample absorbance with the absorbance of pooled plasma enriched with increasing amounts of recombinant human cytokine.

### Plasma nitrate and nitrite levels

NO is a short-lived free radical gas that rapidly reacts with oxygen to generate the stable metabolites nitrate and nitrite. Thus, NO levels were assessed indirectly by measuring the accumulation of nitrates and nitrites. Plasma nitrate and nitrite (NO_x_) determinations were performed using the Griess reaction^27,36^. Results were expressed as μM of NO_x_/sample.

### Statistical analysis

Non-parametric tests were used to assess the comparison of continuous variables because of their non-Gaussian distribution. Values are reported as median and interquartile range (IQR) or percentage as appropriate. Mann–Whitney U and Kruskal–Wallis tests were used to compare two or more groups, respectively, and the-chi-square test to compare proportions. Multivariate Cox proportional hazards models were constructed to assess the relationship of prosthesis loosening and infection with the diverse genotypes and the factors independently associated with such clinical outcomes. All the reported p values are two-sided. A P value < 0.05 was considered as statistically significant. SPSS v. 25 software (IBM Corp., Armonk, NY, USA) was used for statistical calculations. Hardy–Weinberg equilibrium was calculated for each genotype and allele.

### Ethical approval

This study was performed in line with the principles of the Declaration of Helsinki. Approval was granted by the Ethics Committee of the Hospital Universitario Central de Asturias (HUCA).

### Consent to participate

Each participant gave written informed consent for inclusion in the study.

## Results

A total of 339 patients were included in the study: 77 with APL, 117 with PJI, and 145 controls. The median age at the time of the first surgery was 68.8 years (IQR 61.2–74.9), and 60.5% were women. Controls had a median follow-up of their hip or knee implants without developing APL or PJI of 16.46 years (IQR 16.16–16.90). As compared with controls, APL patients were younger and had more hip implants than controls, whereas PJI patients were more commonly males (Table 2).

**Table 2:** Demographic and clinical features of patients with prosthesis loosening, infection and controls.

		Loosening (n = 77)	Infection (n = 117)	Controls (n = 145)	P value
Gender	Female	50 (64.9%)	59 (50.4%)	96 (66.2%)	0.02
	Male	27 (35.1%)	58 (49.6%)	49 (33.8%)	
Age	Years	63.33 (58.40–69.52)	69.06 (61.15–76.00)	70.12 (63.74–75.30)	0.0001
Prosthesis location	Hip	58 (75.3%)	63 (53.8%)	80 (55.2%)	0.001
	Knee	19 (24.7%)	49 (41.9%)	65 (44.8%)	
	Other	0 (0.0%)	5 (4.3%)	0 (0.0%)	
Side	Right	39 (50.6%)	56 (49.6%)	86 (59.3%)	0.2
	Left	38 (49.4%)	57 (50.4%)	59 (40.7%)	
Prosthesis change	Yes	69 (97.2%)	89 (76.1%)	0 (0.0%)	< 0.0001
	No	2 (2.8%)	28 (23.9%)	145 (100%)	
Time to prosthesis change	years	10.08 (4.18–15.04)	4.82 (1.71–9.77)	–	0.0003

The most frequently isolated microorganism was *Staphylococcus epidermidis*, which was recovered alone or in combination with other bacteria in 46 (39.3%) of the PJI patients. Other pure or mixed isolates were *Staphylococcus aureus* in 35 patients (29.9%, 13 of them, 37.1%, methicillin-resistant), other Gram-positive bacteria in 25 (21.4%) and Gram-negative bacteria in 23 (19.7%) patients.

There were no differences in IL-1β levels among the groups, but controls had substantially higher levels of IL-6 than the APL and PJI groups, whereas APL patients had higher TNF-α serum levels than the other two groups. Plasma NOx levels were significantly higher in the controls compared to APL patients (Table 3).

**Table 3:** Cytokine and nitric oxide (NOx) plasma levels in patients with prosthesis loosening, infection and controls.

	Loosening (n = 58)	Infection (n = 99)	Controls (n = 108)	P value
IL-1β (pg/mL)	2.66 (1.77–12.82)	15.58 (2.41–30.17)	2.0 (0.85–3.65)	0.13
IL-6 (pg/mL)	27.94 (8.59–71.53)	30.60 (14.78–65.07)	131.71 (31.00–216.85)	< 0.0001
TNF-α (pg/mL)	30.66 (21.06–45.66)	3.91 (1.66–12.57)	11.96 (0.00–24.61)	< 0.0001
NOx (μM/mL)	36.8 (26.8–51.6)	Not done	98.0 (70–156)	< 0.0001

All genotypes and alleles analyzed were in Hardy Weinberg equilibrium except for the *IL-1α (-889 C/T)* polymorphism in the controls. Table 4 describes the relationships among the diverse polymorphisms analyzed and the three groups of patients. Overall, there were no statistically significant associations when the three groups were considered altogether. However, when compared individually, the genotypes and alleles of the *IL-1β (*+*3954 C/T, exon 5, rs1143634)* polymorphisms were differently distributed (P = 0.03 and P = 0.02, respectively) between APL patients and controls, mainly due to a higher representation of the variant *T* allele and *TT* genotype in the APL group (these individual comparisons data are not shown in Table 4).

**Table 4:** Polymorphisms of *IL-1α, IL-β, TNF-α, NOS3 and NOS2* in patients with aseptic loosening, infection and controls.

Gene polymorphism	Genotype	Loosening n (%)	Infection n (%)	Control n (%)	P value	Alleles	Loosening n (%)	Infection n (%)	Control n (%)	P value
*IL-1α (-889 C/T)*	*CC*	28 (45.2)	54 (52.4)	61 (48.8)	0.2	*C*	85 (68.5)	146 (70.9)	181 (72.4)	0.7
	*CT*	29 (46.8)	38 (36.9)	59 (47.2)		*T*	39 (31.5)	60 (29.1)	69 (27.6)	
	*TT*	5 (8.0)	11 (10.5)	5 (4.0)						
*IL-1β (*+ *3954 C/T)*	*CC*	34 (44.2)	66 (56.9)	81 (55.9)	0.1	*C*	102 (66.2)	174 (75.0)	221 (76.2)	0.06
	*CT*	34 (44.2)	42 (36.2)	59 (40.7)		*T*	52 (33.8)	58 (25.0)	69 (23.8)	
	*TT*	9 (11.6)	8 (6.9)	5 (3.4)						
*TNF-α (-308 G/A)*	*GG*	47 (75.8)	86 (74.1)	83 (66.4)	0.4	*G*	109 (87.9)	200 (86.2)	204 (81.6)	0.2
	*GA*	15 (24.2)	28 (24.1)	38 (30.4)		*A*	15 (12.1)	32 (13.8)	46 (18.4)	
	*AA*	0 (0.0)	2 (1.7%)	4 (3.2%)						
*NOS3 (-786 T/C)*	*TT*	21 (33.9)	29 (28.2)	46 (36.8)	0.3	*T*	73 (58.9)	114 (55.3)	143 (57.2)	0.8
	*TC*	31 (50.0)	56 (54.4)	51 (40.8)		*C*	51 (41.1)	92 (44.7)	107 (42.8)	
	*CC*	10 (16.1)	18 (17.5)	28 (22.4)						
*NOS2 (*exon 22)	*GG*	18 (29.0)	34 (33.0)	47 (37.6)	0.7	*G*	72 (58.1)	121 (58.7)	155 (62.0)	0.7
	*GA*	36 (58.1)	53 (51.5)	61 (48.8)		*A*	52 (41.9)	85 (41.3)	95 (38.0)	
	*AA*	8 (12.9)	16 (15.5)	17 (13.6)						

Plasma IL-1β and IL-6 were significantly higher in APL *IL-1β* *(*+*3954C/T, exon 5) T* allele carriers compared to non-carriers (IL-1 β: 11.79 [IQR 3.41–21.14] vs. 2.11 [IQR 1.61–5.5], P < 0.03); IL-6: 43.59 [IQR 16.21–146.47 vs. 38.12 (IQR 15.0–128.7, P = 0.006]). Plasma levels of NOx did not differ in carriers of the different *NOS3* and *NOS2* genotypes.

Multivariate Cox proportional hazard models were elaborated for each polymorphism to evaluate the associations between the diverse genotypes with prosthesis loosening and infection over time. No polymorphisms genotypes were significantly associated with PJI, but genotypes of the *IL-1β (*+*3954 C/T, exon 5)* and *NOS2 (exon 22)* SNPs showed different behavior in the APL group. Table 5 shows the results of these two multivariate regressions. *IL-1β* *(*+ *3954 C/T, exon 5)* SNP independently associated with knee prosthesis while the *NOS2 (exon 22)* SNP also associated with knee prosthesis and male gender.

**Table 5:** Factors independently associated with prosthesis loosening according to the multivariate Cox regression.

	Factor	HR (95% CI)	P value
*IL-1β (*+*3954C/T)* model	Knee prosthesis	2.488 (1.307–4.739)	0.005
	*IL-1β (*+*3954 C/T)* SNP	–	0.012
	*TT* vs. *CC* genotype	3.704 (1.274–10.753)	0.016
	*TT* vs. *CT* genotype	4.587 (1.675–12.500)	0.003
*NOS2 (exon22)* model	Knee prosthesis	4.367 (1.972–9.709)	0.0003
	Male gender	2.252 (1.121–4.525)	0.023
	*NOS2 (exon22)* SNP	–	0.048
	*AA* vs. *GG* genotype	3.509 (1.266–9.709)	0.016
	*AA* vs. *GA* genotype	2.639 (1.072–6.494)	0.035

**Figure 1:**
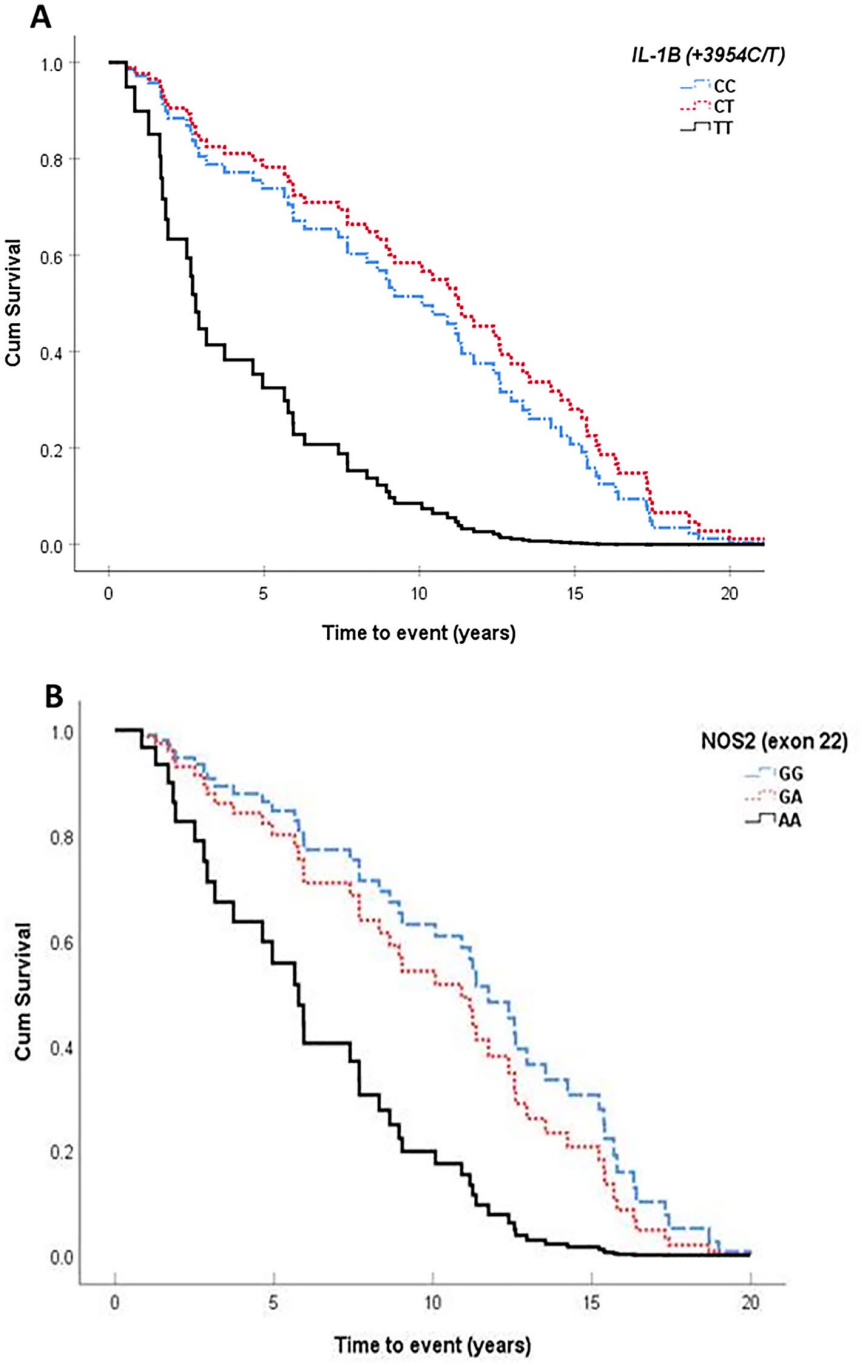
Cox regression curves comparing the development over time of aseptic prosthetic loosening (APL), according to the *IL-1β (*+*3954 C/T)* (**A**) and *NOS2 (exon22)* (**B**) genotypes.

Figure 1 depicts the Cox regression curves survival of the joint replacement according to the *IL-1β (*+*3954 C/T)* and *NOS2 (exon 22)* genotypes. Patients carrying the respective variant homozygous genotypes experienced earlier prosthesis loosening than those carrying the heterozygous and homozygous wild genotypes, an effect that was mainly noted during the first 5 years after surgery.

## Discussion

Total hip and knee replacements are some of the most successful and cost-effective surgeries performed. Although the success rate at 10 years exceeds 90% in hip^37,38^ and knee arthroplasties^39–43^, prosthesis, failure remains a problem necessitating revision surgery, which is costly both in monetary terms and morbidity^42,43^. It is difficult to predict who will require an early replacement.

We found a significant association between a polymorphism within exon 5 of *IL-1β (*+*3954 C/T, rs1143634)* and early APL. The *TT* genotype of this *IL-1β* polymorphism showed a 3.7-fold higher probability of requiring a joint replacement because of loosening within the first 5 years after surgery, compared to controls with implants lasting ≥ 16 years. This association between *IL-1β* polymorphism and early loosening association was especially strong with knee APL (Table 5).

*IL-1* and *TNF-α* genes are transcriptionally activated in patients and in experimental models of APL^44–47^. IL-1 and TNF-α activate bone-resorbing giant cells that can increase bone loss around implants, which is characteristic of APL^48^. Osteoporotic fractures due to a reduction in the bone mineral density are associated with an 86-base pair repeat polymorphism in the *IL-1RN* gene^49^. There is a linkage disequilibrium between *IL-1α, IL-1β* and *IL-1RN* genes, all of which are encoded very close to each other on the long arm of chromosome 2^50^. Thus, it is difficult to be sure whether these associations are specific for a particular gene in the IL-1 family, or even depend on other unknown gene from the same chromosome. Some haplotypes of the *IL1R1*-*IL1A-IL1B-IL1RN* gene cluster associated with enhancement to (*IL1A-IL1B-IL1RN* haplotype) or protection against knee osteoarthritis (*IL1B-IL1RN* haplotype)^20^.

Associations between polymorphisms in the *IL-1* gene and aseptic loosening have been studied in maxillofacial surgery. An association between carriage of the *IL-1β (*+*3954 C/T, exon 5) T* allele and other IL-1 polymorphisms and unsuccessful retaining overdentures and periodontitis in smokers and non-smokers was reported^21,51–56^. An association between a *(-889)* polymorphism of the promoter region of *IL-1α* and susceptibility to juvenile rheumatoid arthritis and to osteomyelitis was also reported^11,18^.

The *IL-1* gene variations were previously examined for associations with various chronic inflammatory diseases and with altered levels of inflammatory mediators^56,57^. We observed that APL patients with the *TT* genotype of the *IL-1β* polymorphism had the highest plasma levels of IL-1β and IL-6 compared to carriers of other genotypes of the APL group. Although immunohistochemistry studies of the removed synovial membranes or measure of the cytokine levels in synovial fluid were not performed in our study, based on the work of others^58^ we would expect that patients with the *TT* genotype of the *IL-1β* polymorphism might have proinflammatory cytokines (IL-1β and IL-6) overexpressed in synovial fluids around the implants, facilitating the aseptic loosening of the prosthesis.

We also found an association of the *NOS2 (exon 22)* polymorphisms with early APL. Carriers of the *AA* genotype and *A* allele of this *NOS2* polymorphism had a 3.5 fold higher probability of early loss of their implants, mainly during the first 5 years after surgery. The association of *NOS2 (exon 22* polymorphism with APL was especially strong with knee prosthesis loosening and with male gender.

Although plasma NOx levels were significantly decreased in APL patients compared to controls, we could find no differences in plasma NOx levels among carriers of the different genotypes of the *NOS2* and NOS3 polymorphisms. However we have measured plasma NOx only at one point in time, rather than a 24 h urine nitrate assay that would give a better picture of how much NOx is produced. A relationship of the *NOS3 (27-bp, intron 4)* polymorphism and osteomyelitis^27^ and of the *NOS3(Glu298Asp)* polymorphism and hip osteoporotic fractures^28^ was previously reported emphasizing the key role of NO in bone metabolism.Thus, the mechanism underneath the association of this *NOS2 (exon 22)* polymorphism and APL remain elusive.

Regarding PJI, no association of cytokines or *NOS* polymorphism were observed in our study. An association of the other IL-1 *β* genetic variant, the *IL-1β (-511C/T, rs 16944)* polymorphism, not genotyped here, and PJI was reported^32,33^. However the *TNF-α (-308 G/A rs1800629)* polymorphism negative association with PJI we observed fully agree with others^59^.

The main limitations of this study is the relatively small number of APL patients included and the limited number of cytokines and *NOS2* and *NOS3* polymorphisms genotyped due to budget limitations. The strong points of the work are the novelty of genotyping *NOS*2 and *NOS3* polymorphisms, the inclusion of a good number of PJI patients and the use of patients with orthopaedic prosthesis lasting > 16 years as controls.

To our knowledge this the first study describing an association of *IL-1β* gene *(*+*3954 C/T, exon 5, rs1143634)* and *NOS2 (exon 22)* polymorphisms and early APL. However, further research in larger European and non-European populations is needed to clarify these genetic associations with early APL.

## Data Availability

All data generated or analysed during this study are included in this published article.
