# Author Correction: *IL-1 β* gene (+ *3954 C/T,* exon 5, rs*1143634*) and *NOS2 (exon 22)* polymorphisms associate with early aseptic loosening of arthroplasties

**DOI:** 10.1038/s41598-022-26126-w

**Published:** 2022-12-13

**Authors:** Esteban López‑Anglada, Julio Collazos, A. Hugo Montes, Laura Pérez‑Is, Imanol Pérez‑Hevia, Sergio Jiménez‑Tostado, Tomás Suárez‑Zarracina, Victoria Alvarez, Eulalia Valle‑Garay, Víctor Asensi

**Affiliations:** 1 Traumatology Department, Hospital Universitario Central de Asturias, Oviedo, Spain; 2 Infectious Diseases Section, Hospital de Galdacano, Vizcaya, Spain; 3 Biochemistry and Molecular Biology Department, University of Oviedo School of Medicine, Oviedo, Spain; 4 Infectious Diseases Unit, Infectious Diseases Section, Hospital Universitario Central de Asturias, University of Oviedo School of Medicine, Avda Roma S/N, 33011 Oviedo, Spain; 5 Molecular Genetics Section, Hospital Universitario Central de Asturias, Oviedo, Spain; 6 Group of Translational Research in Infectious Diseases, Instituto de Investigación Sanitaria del Principado de Asturias (ISPA)., Oviedo, Spain

Correction to: *Scientific Reports* 10.1038/s41598-022-22693-0, published online 01 November 2022

The original version of this Article contained errors in reference citations in the Discussion section.

*IL-1* and *TNF-α* genes are transcriptionally activated in patients and in experimental models of APL^44–48^. IL-1 and TNF-α activate bone-resorbing giant cells that can increase bone loss around implants, which is characteristic of APL^49^. Osteoporotic fractures due to a reduction in the bone mineral density are associated with an 86-base pair repeat polymorphism in the *IL-1RN* gene^50^. There is a linkage disequilibrium between *IL-1α, IL-1β* and *IL-1RN* genes, all of which are encoded very close to each other on the long arm of chromosome 2^51^. Thus, it is difficult to be sure whether these associations are specific for a particular gene in the IL-1 family, or even depend on other unknown gene from the same chromosome. Some haplotypes of the *IL1R1*-*IL1A-IL1BIL1RN* gene cluster associated with enhancement to (*IL1A-IL1B-IL1RN* haplotype) or protection against knee osteoarthritis (*IL1B-IL1RN* haplotype)^20^. Associations between polymorphisms in the *IL-1* gene and aseptic loosening have been studied in maxillofacial surgery. An association between carriage of the *IL-1β (*+ *3954 C/T, exon 5) T* allele and other IL-1 polymorphisms and unsuccessful retaining overdentures and periodontitis in smokers and non-smokers was reported^21,52–56^.

now reads:

*IL-1* and *TNF-α* genes are transcriptionally activated in patients and in experimental models of APL^44–47^. IL-1 and TNF-α activate bone-resorbing giant cells that can increase bone loss around implants, which is characteristic of APL^48^. Osteoporotic fractures due to a reduction in the bone mineral density are associated with an 86-base pair repeat polymorphism in the *IL-1RN* gene^49^. There is a linkage disequilibrium between *IL-1α, IL-1β* and *IL-1RN* genes, all of which are encoded very close to each other on the long arm of chromosome 2^50^. Thus, it is difficult to be sure whether these associations are specific for a particular gene in the IL-1 family, or even depend on other unknown gene from the same chromosome. Some haplotypes of the *IL1R1*-*IL1A-IL1BIL1RN* gene cluster associated with enhancement to (*IL1A-IL1B-IL1RN* haplotype) or protection against knee osteoarthritis (*IL1B-IL1RN* haplotype)^20^. Associations between polymorphisms in the *IL-1* gene and aseptic loosening have been studied in maxillofacial surgery. An association between carriage of the *IL-1β (*+ *3954 C/T, exon 5) T* allele and other IL-1 polymorphisms and unsuccessful retaining overdentures and periodontitis in smokers and non-smokers was reported^21,51–56^.

The original Article has been corrected.

